# Structural and mechanistic fundamentals for designing of cellulases

**DOI:** 10.5936/csbj.201209006

**Published:** 2012-10-16

**Authors:** Sandro R. Marana

**Affiliations:** a Departamento de Bioquímica, Instituto de Química, Universidade de São Paulo, CP 26077, São Paulo, 05513-970, SP, Brazil

**Keywords:** cellulose hydrolysis cellulose saccharification, cellulose, cellulase, cellobiohydrolase

## Introduction

Lignocellulosic biomass has been pointed as a promising source of renewable and sustainable energy. However the harvesting of this energy depends on several steps among them the hydrolysis of the cellulosic component. Due to the specificity, enzymatic hydrolysis is an interesting option to accomplish such task [1]. That has prompted the selection of natural cellulases and also the development of “new” cellulases exhibiting properties designed for this process. However, the lack of substrates that appropriately simulate the natural hydrolysis conditions in high-throughput assays has hindered the development of cellulases based on directed-evolution [2]. Hence, the “rational designing” of cellulases based on solid structural and mechanistic data is the main option to tackle this question.

## Structural and functional properties of the noncomplexed cellulolytic systems

Noncomplexed cellulolytic systems are composed by isolate enzymes that work cooperatively and synergistically to accomplish the hydrolysis of crystalline cellulose. The most studied noncomplexed cellulolytic system is produced by the fungus *Trichoderma reesei* (also known as *Hypocrea jecorina*). Such system is composed by endocellulases, processive endocellulases and processive exocellulases [3]. But the majoritary components are two enzymes denominated TrCel7A and TrCel6A (formely CBH I and II, respectively), which together correspond to more than 50% of the cellulolytic enzymes produced by *T. reesei*. The system is completed by the endocellulases TrCel7B, TrCel5A and TrCel12A, which are produced in smaller amount and help the action of the TrCel7A and TrCel6A by increasing the number of chain ends in the cellulose [4].

**Figure 1:**
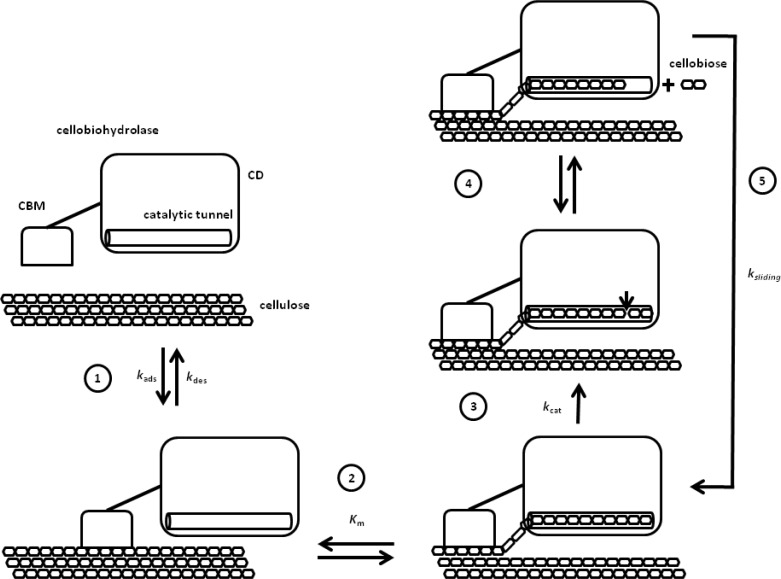
**Schematic steps of the crystalline cellulose hydrolysis by cellobiohydrolases**. Step 1 – Interaction with the cellulose surface mediated by the CBM. Rate constants of adsorption (*k*_ads_) and desorption (*k*_des_) are related to this step. Step 2 – Introduction of a single cellulose chain into the catalytic tunnel of the CD and formation of the productive complex. A *K*_m_ may be associated to this complexation. Step 3 – Hydrolysis of the glycosidic bond. Step 4 – Release of the product, cellobiose, which may diffuse back into the active site inhibiting the cellobiohydrolase. The affinity for cellobiose is expressed by a *K*_i_. Step 5 – Sliding of the cellulose chain forming a “new” productive complex. In the processive activity of the cellobiohydrolases the steps 3, 4 and 5 are repeated many times without dissociation from the cellulose chain.

TrCel7A and TrCel6A are processive enzymes, so when associated to a single cellulose chain they go through repeated catalytic cycles hydrolyzing multiple glycosidic bonds and producing cellobiose as products. TrCel7A attacks the cellulose chain at the reducing end, whereas TrCel6A starts from the non-reducing end. Both enzymes have a two-domain architecture composed by a catalytic domain (CD) and a carbohydrate binding module (CBM) [5–8]. In spite of some particularities, these enzymes follow the same general steps when hydrolyzing crystalline cellulose (Figure 1). The first step is the binding to the hydrophobic face of the crystalline cellulose mediated by the CBM [9–11]. Then a single cellulose chain is detached from the cellulose surface, which include the disruption of the intra- and inter-chain hydrogen bonds of this substrate, and introduced in the active site of the CD forming a productive complex. The combination of these steps takes about 10 s for TrCel7A [12]. Following that the β-glucosidic bond is cleaved, a reaction in which the *k*_cat_ is about 2 to 4 s^-1^ for TrCe7A [12–14] and 14 s^-1^ for TrCel6A [15]. After that the product, cellobiose, is released from the cellobiohydrolase active site. The next step is the sliding of the cellulose chain to occupy the empty product subsites forming a “new” productive complex [6–8]. The repetition of the steps 3, 4 and 5 defines the processive action of TrCel7A and TrCel6A. Experimental determinations using bacterial crystalline cellulose indicate that the processivity of TrCel7A is around 60 cellobiose units, whereas its theoretical upper limit is 4000 [12, 13]. Thus, considering the observed processivity and *k*_cat_, TrCel7A should complete a processive run in about 30 s [12]. The dissociation of TrCel7A from the crystalline cellulose surface after the product release is very slow, taking about 24 min [13]. Therefore the recruitment of TrCel7A for new “processive runs” is a critical step for crystalline cellulose hydrolysis.

**Figure 2:**
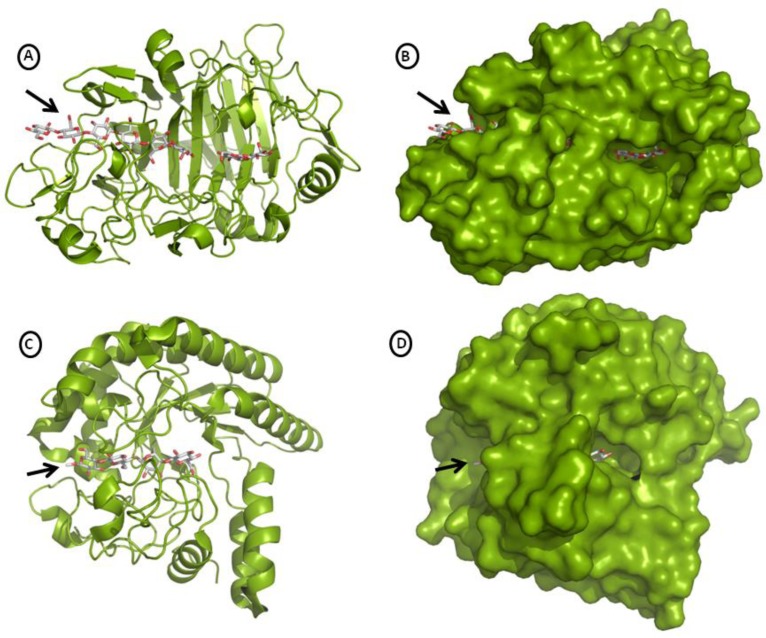
**Structures of the catalytic domain of TrCel7A and TrCel6A**. **A)** Top view of TrCel7A. Cellotetraose and cellobiose are bound to the active site. The entrance of the tunnel is indicated by an arrow. **B)** Space filling model of TrCel7A (top view) showing that the cellodextrin chain is totally embraced by the tunnel-shaped active site. The entrance of the tunnel is indicated by an arrow. Cellobiose is viewed at the tunnel exit. **C)** Top view of TrCel6A. The active site on the top of the barrel is filled with cellotetraose. The entrance of the tunnel is indicated by an arrow. **D)** Space filling model of TrCel6A showing that the cellodextrin is totally enclosed in the tunnel-shaped active site. A small part of the cellotetraose chain end is observed at the tunnel exit. Structures were based on the PDB files 7CEL and 1QK2, respectively, and visualized using the software PyMol v0.99 (DeLano Scientific LLC).

The catalytic domain of TrCel7A, classified in the Family 7 of the Glycoside Hydrolase [16], is formed by 434 amino acid residues organized as two antiparallel β sheets that are stacked forming a curved β sandwich. The loops segments connecting the β strands from the convex face of the β sandwich are short, but those from concave face are longer, specially the called “exo-loop” formed by residues 243-256, and form a tunnel (50 Å) that runs along that face and contains the active site (Figure 2). The CD has three sites (N45, N270 and N384) of *N*-glycosylation, each of them linked to a single N-acetylglucosamine residue [17]. The CBM of TrCel7A belongs to the family 1 [18], contains only 36 amino acid residues which are organized in three antiparallel β strands connected to the CD by a short linker which is 28 amino acid residues long [7, 8]. All eight threonine residues found in the linker are glycosylated with one up to three mannoses, whereas three serine residues are glycosylated with a single mannose [17]. The *O*-glycosylation pattern of the linker is affected by growth conditions and host expressing TrCel7A [19, 20].

The catalytic domain of TrCel6A (364 amino acid residues; classified in the Family 6 of the Glycoside Hydrolase) is a distorted α/β barrel composed by seven parallel strands that exhibits a 20 Å long tunnel enclosed by two loops (residues 172 to 189 and 394 to 429) at the C-terminal top of the barrel. Two disulfide bonds (C176-C235 and C368-C415) stabilize these loops delimiting the active site (Figure 2). The CBM of TrCel6A is homologous to TrCel7A, but it is connected to the N-terminal of the CD by the *O*-glycosylated linker containing 30 amino acid residues [6]. The CD is N-glycosylated at sites N310, where a high mannose glycan (7-9 residues) is found, and N289, which is linked to a single N-acetylglycosamine. Nevertheless, variation of this glycosylation pattern was observed. The *O*-glycosylation at the linker range from 39 to 46 residues, which are connected at threonine residues 87 and 97 and serine 106, 109, 110 and 115 [21]. The two loops that enclose the active site of TrCel6A are known to alternate between opened and closed positions, allowing the initiation of the catalytic activity to occur at internal bonds of cellulose in addition to the chain ends [22–24].

For TrCel7A the probability of endo-mode initiation on crystalline cellulose is 0.41. As a comparison, for cellulases that do not have loops covering the active site and are considered strict endo-mode, like TrCel5A, the probability of endo-initiation is 0.97. In agreement, a short exo-loop covering the active site, as observed in the cellobiohydrolase I PcCel7D from the fungus *Phanerochaete chrysosporium* [25], determines a higher probability (0.88) of endo-mode initiation [13]. Additionally, the deletion of the TrCel7A exo-loop increased its endocellulase activity [26].

Regardless the endo-or exo-initiation, the CBM is essential for the initial interaction to the cellulose surface. Actually in one of the flat faces of the CBM three tyrosines (Y5, Y31 and Y32) and two polar residues (Q7 and N29) are positioned to interact through hydrogen bonds with the hydrophobic face of the crystalline cellulose [5, 10]. Site-directed mutagenesis studies showed that the replacement of those residues by alanines decreases the affinity of TrCel7A for crystalline cellulose [27, 28]. Atomic force microscopy experiments showed that the isolated CBM from TrCel7A slides unidirectionally on the surface of the cellulose at 3.5 nm/s. A similar velocity was determined for the advance of the complete TrCel7A in processive activity [29]. Considering that cellobiose is about 1 nm long, such rate is compatible with the *k*_cat_ (0.3 s^-1^) observed for TrCel7A [13]. Molecular dynamics studies indicated that during the sliding the “interaction face” of the CBM is parallel to the cellulose surface and that the CBM treks preferentially on a single cellulose chain, so its lateral diffusion is not significant [30]. Additionally, the CBM sliding on the cellulose surface exhibits peaks of highest affinity at each 1nm, which corresponds to the length of the cellobiose, the main product of TrCel7A and TrCel6A catalytic activity [31]. So after each cellobiose release, the sliding of the CBM is precisely enough to fill again the tunnel of the CD. Molecular simulations showed that the linker connecting the CBM to the CD is highly flexible, a property which is not changed by the *O*-glycosylation. Nevertheless the most likely conformation of the glycosylated linker is 16Å longer than the non-glycosylated one (53 Å *versus* 37 Å). Due to the linker flexibility, the CD may search for the cellulose chain end within a maximum range of 8 cellobiose units while the CBM interacts with the cellulose surface [32].

The introduction of the isolate chain of cellulose into the tunnel of the CD is mediated by interactions with the W40 in TrCel7A and W272 in TrCel6A, residues that are positioned close to the opening of the catalytic tunnel. Indeed, the replacement of W40 by A impairs the sliding of TrCel7A on the crystalline cellulose [29]. Additionally, the replacement of W272 by A and D decreases the TrCel6A activity upon crystalline cellulose whereas the binding to this substrate, probably mediated only by the CBM, is not altered [33]. In agreement, molecular dynamics simulations showed that the deletion of W272 side chain reduced the affinity of TrCel6A for the cellulose chain and increased its fluctuation inside the active site [34].

Once TrCel6A and TrCel7A are associated to the cellulose surface and the productive complex is formed, additional steps of sliding depends on the hydrolysis of the glycosidic bond, cellobiose release and translocation of the substrate inside the catalytic tunnel. Indeed, an inactive mutant TrCel7A, which has a single replacement of the catalytic residue E212, binds cellulose, but does not slide on its surface [29].

The tunnel that encloses the active site of TrCel7A is divided in 10 subsites (-7 to +3), each one binds one glycosyl unit. An external subsite (+4) is positioned close to the tunnel exit. Based on the mechanisms described above the reducing end of a single cellulose chain is introduced into the TrCel7A active site filling sequentially the subsites from -7 to +3 and forming a productive complex. The hydrolytic cleavage of the glycosidic bond occurs between the subsites -1 and +1 when the glycosidic oxygen is pointing towards the residue E217, the catalytic acid. Considering that the glycosyl units of a cellulose chain alternate their orientation in 180°, after each bond cleavage and product release the chain has to be moved two subsites ahead (filling again subsites +1 and +2) in order to place the glycosidic bond in scissile orientation again. That explains the processive release of cellobiose as the main product of the TrCel7A activity. The hydrolytic reaction catalyzed by TrCel7A follows a double-displacement mechanism that depends on E212 and E217 as catalytic nucleophile and acid, respectively. The configuration of the anomeric carbon of the produced cellobiose is β, so TrCel7A is a retaining glycosidase. Four tryptophan residues, W40, W38, W367 and W376 distributed along the tunnel of TrCel7A are determining structural elements of the subsites -7, -4, -2 and +1. The indole group of their side chains forms stacking interactions with the β or α face of the glucosyl units. Hydrogen bonds mediated by water molecules are also formed between the substrate and subsites -7 to -2. The stacking interactions, which do not have a strong directional component, and indirect hydrogen bonds mediated by water favor the sliding of the cellulose chain during the processive cycles of TrCel7A. Interestingly, in the subsites -3 and -2, hydrogen bonds involving charged residues and the stacking interactions with W38 and W367 stabilizes a twisted conformation of the cellulose chain favoring the distortion of the ring of the glucosyl unit that interacts with the subsite -1. Such distorted conformation resembles the proposed transition state for the reaction catalyzed by TrCel7A. Five residues, the majority of them charged, interact with the cellulose chain in the subsite +1, whereas R394 interacts with the glucosyl unit in the subsite +2. The interactions formed by the subsites +1 and +2 are important for the productive binding of the substrate [7, 8].

The shorter tunnel of TrCel6A is divided in 4 subsites (-2 to +2), whereas an extra subsite (+4) is positioned at its opening. On the other hand, cellobiohydrolase II from *Chaetomium thermophilum* and *Humicola insolens* have larger catalytic tunnels exhibiting 7 (-3 to +4) and 8 subsites (-4 to +4), respectively [24, 35]. The non-reducing end of a single cellulose chain fills sequentially the tunnel from the subsite +4 to -2. Interestingly as observed for TrCel7A the cellulose chain also undergoes a twist in the subsites (+3 and +4) placed before the cleavage position, which also occurs between subsites +1 and -1 with the participation of the residues D175 and D221, which are connected by a hydrogen bond and positioned in the same side of the scissile bond. In the hydrolytic reaction D221 acts as a catalytic acid promoting the protonation of the glycosidic oxygen, whereas the negatively charged D175 contributes to the electrostatic stabilization of the transition state and also accepts a proton from a chain of two water molecules that makes a nucleophilic attack on the anomeric carbon of the glycosidic bond. As in this reaction the configuration of the anomeric carbon is changed to, TrCel6A is called an inverting glycosidase [15].

The general architecture of the binding subsites of the catalytic tunnel of TrCel6A resembles that of TrCel7A. Tryptophan residues W135, W367, W269 and W272 form platforms for stacking interactions with the glycosyl units of the cellulose chain in the subsites -2, +1, +2 and +4, respectively. Polar and charged residues are also present and form hydrogen bonds with the glycosyl units [6]. Structural analysis of HiCel6A revealed that the binding of cellodextrin chain at the intermediate position of the translocation route to the product subsites is mostly mediated by water, whereas the productive complex is sustained by direct interactions. Additionally movements of secondary structure elements alter the positioning of the subsite platforms during the sliding process, whereas the catalytic acid (D266) alternates between positions close and apart from the substrate. Such structural dynamic seems to be the basis for the cellulose chain sliding through the catalytic tunnel of HiCel6A [24].

Considering the residues forming the subsites -7 to -1 of TrCel7A there is a trend to increase the number of the interactions with the cellulose chain along the catalytic tunnel, specially at the subsites +1 and +2 [8]. Such putative affinity gradient could favor the sliding of the cellulose chain into the subsites +1 and +2 during the repeated cycles of the processive action of TrCel7A upon crystalline cellulose. Indeed the deletion of the residues 245 to 252 of the exo-loop, which roofs the subsites +2 and +1, reduces to half the processivity of TrCel7A upon crystalline cellulose, whereas the activity upon amorphous cellulose is not affected [36]. In agreement the comparison of TrCel7A from different organisms indicates that shorter loops covering the subsites +1 and +2 determine a lower potential processivity upon crystalline cellulose [13]. Also, the binding free energy for cellobiose in the subsites +1 and +2 is very different for processive and non-processive cellulases. Actually, computational simulations indicate that the binding of cellobiose to TrCel7A is about 5 *k*cal/mol stronger than to TrCel7B, a homologous non-processive endocellulase [37].

In agreement to the discussed above for TrCel7A, molecular dynamics simulations of the interaction between TrCel6A and cellodextrins indicated the presence of an increasing affinity gradient in the direction of the subsites that bind the terminal cellobiose. The relative binding free energies are 3.8 *k*cal/mol in the subsite +4, -1.3 *k*cal/mol in the subsite +2, 1.6 *k*cal/mol in the subsite +1 and 9.8 *k*cal/mol in the subsite -2 [34]. The estimate for subsite -2 is in accordance with the binding free energy of cellobiose to the product subsites (-2 and -1) of TrCel6A (13.9 *k*cal/mol), whereas a similar interaction is proposed for TrCel7A (10.9 *k*cal/mol) [37]. Interestingly the interaction in the product subsites is enough to remove one cellobiose unit from the surface of the crystalline cellulose [38], a further suggestion that the binding to the product subsites could propel the sliding of the cellulose chain during the processive action of TrCel7A and TrCel6A.

However, a side effect of the higher affinity at the product subsites +1 and +2 is the inhibition of TrCel7A by its product, cellobiose, which after released may diffuse back into those subsites blocking the processive action of the enzyme. Indeed, the deletion of the exo-loop forming the roof of the subsites +1 and +2 of TrCel7A resulted in a dramatic decrease in the inhibition by cellobiose, the *K*_i_ increased about 10 times [36]. In agreement molecular dynamics simulations indicate that point mutations at the subsite +1 reduce in 50% the affinity of TrCel7A for cellobiose [39]. The inhibition of TrCel7A by cellobiose is mixed type, so involves the formation of a ternary complex (ESI), in which a cellulose chain isolated from the cellulose surface is bound to the subsites -1 to -7 while a cellobiose occupies the subsites +1 and +2 blocking the processive advance of TrCel7A [40]. A complex EI, TrCel7A-cellobiose, is also formed, probably preventing the enzymes from engaging in the cellulose hydrolysis. The *K*_i_ for the inhibition of crystalline cellulose hydrolysis by TrCel7A is 1.6 mM [40].

Thus, although a general view of the processive action of TrCel7A is available, due to the multiplicity and complexity of its steps further functional details remain to be uncovered. Computational methods are potentially important players to tackle these unanswered questions [41]. Particular steps in need of a deeper description are the thermodynamics of the participation of the CBM and CD in favoring the initial cellulose decrystallization process, the structural dynamics of the productive complex including the bond breaking and expulsion of the product and finally the energetics of the cellulose chain sliding into the product subsites for initiation of a new activity cycle.

## Kinetics of cellulose hydrolysis

A noteworthy characteristic of the enzymatic hydrolysis of cellulose is the reduction of the rate along the time. This behavior is observed for hydrolysis performed with isolate TrCel7A and also for mixtures containing TrCel7A and endoglucanases (TrCel7B). Several kinetics models have tried to simulate that behavior by incorporating parameters and equations related to the mechanisms described in the functional and structural properties of TrCel7A [42]. For instance a recent mechanistic model [43] features kinetic parameters for the CBM binding to and desorption from crystalline cellulose (*k*_ads_ and *k*_des_), the formation of the productive complex by introducing the cellulose chain into the catalytic tunnel of the CD (*K*_m_), the cleavage of the glycosidic bond (*k*_cat_) and the cellobiose inhibition (*K*_i_). Additionally, crystalline cellulose properties as the degree of polymerization for different chains and accessible superficial area of cellulose along the reaction time were also incorporated. In this direction models incorporating detailed evolution of cellulose morphology along the hydrolysis have been developed showing its effect on the slowing down of the hydrolysis rate [44–46]. Simulations using a mechanistic model showed that the cellobiose inhibition of TrCel7A decreases the rate of cellulose hydrolysis along the reaction time. However, only a *K*_i_ on the micromolar range, an affinity much higher than that experimentally observed, would entirely explain the rate decrease detected in experiments [43]. On the other hand, these simulations also revealed that the rate limiting step in the cellulose hydrolysis by TrCel7A is the productive binding of the cellulose chain (*K*_m_), a step related to the introduction and sliding of the chain into the catalytic tunnel of the CD. In agreement, *k*_cat_ changes did not affect the rate of the process, confirming that the hydrolysis of the glycosidic bond is not the rate limiting step.

These simulations agree to the conclusions drawn from enzyme kinetics experiments. Based on them it has been proposed that once associated to the cellulose, TrCel7A moves ahead catalyzing multiple bond cleavage until it encounters an obstacle and gets blocked [13, 47, 48]. Due to a low dissociation constant, the enzyme remains stuck in an unproductive complex. Hence the concentration of productive TrCel7A decreases reducing the rate of hydrolysis [13, 48]. Such stacking of TrCel7A at obstacles on the cellulose was directly observed by atomic force microscopy, showing that the “traffic” of several TrCel7A is simultaneously blocked leading to “jams” on the cellulose surface [49]. This proposal is also supported by the observation that the rate of cellulose hydrolysis is not affected by the ratio of cellulose conversion and that the concentration of TrCel7A bound to cellulose does not decrease along the reaction [13]. Indeed, the decrease of the productive TrCel7A would result in an apparent increase of the *K*_m_, which is in agreement to the simulation outcome showing that the binding of the cellulose chain in the catalytic tunnel of the CD is the parameter that significantly affects the rate of hydrolysis [43]. The so-called synergism between cellobiohydrolases and endoglucanases (exo-endo) and also between TrCel6A and TrCel7A (exo-exo) may also be interpreted in the light of the proposal that obstacles block TrCel7A sliding. Hence, endoglucanases could cut the cellulose chain prior and after an obstacle, producing points for TrCel7A release and also creating points for re-initiation of its processive run [13]. Additionally considering that due to the more flexible loops covering the active site TrCel6A exhibits a higher probability of endo-mode initiation, this cellobiohydrolase could have an action similar to the endoglucanases explaining the synergism between TrCel6A and TrCel7A [49, 50]. This proposal is in agreement with earlier observations that simultaneous action of TrCel6A and TrCel7A is not a precondition for synergism, which is also observed even when crystalline cellulose is treated with TrCel6A previously to incubation with TrCel7A [51].

Therefore, the overall picture suggests that catalytic tunnel of TrCel7A is a key to isolate a single chain from the cellulose surface impeding its re-crystallization. Once committed with a single chain, the enzyme has to proceed processively. However, the catalytic tunnel impedes the enzyme to dissociate from the cellulose chain when blocked by an obstacle, resulting in reduction of the rate along the reaction time [13, 48]. Considering that, the participation of endocellulases in the noncomplexed cellulolytic systems is not restricted to the production of chain ends for initiation of hydrolysis, but they also contribute forming release points for cellobiohydrolases avoiding the stacking at obstacles. Moreover, the synergistic action of TrCel7A and TrCel6A may also be related to the endo-initiation by TrCel6A [12, 49, 50].

## Summary and Outlook

The determination of the three-dimensional structures of TrCel7A and TrCel6A on the 90's revealing a bimodular structure containing a CBM and a CD featuring a tunnel-shaped active site set up the framework for the functioning of these cellobiohydrolases. The tunnel-shaped active site embraces a single cellulose chain, isolated from the crystal surface by means of the CBM, hinders its recrystallization and processively removes cellobioses from the chain end. As the interactions with the CBM contributes only for the initial binding, the affinity of the cellulose chain for the product subsites in the catalytic tunnel, which have to be filled again after each catalytic cycle, seems to be the potential for propelling the cellobiohydrolase during the processive action. However, it is not clear how the cellulose chain sliding is triggered, leaving its stable position within the subsites prior to the cleavage point and forming a “new” productive complex. Internal motions of the cellobiohydrolase-cellulose complex are probably involved, but it is not clear the source of the power to overcome the barrier to initiate the translocation. Additionally it is not clear the participation of the CBM and the linker connecting the CBM to the CD on the sliding.

The tunnel-shaped active site is the key to keep the cellulose chain away from the crystal surface and ensure the processive activity of the cellobiohydrolase. But, the tunnel also impedes the dissociation from the cellulose when the cellobiohydrolase gets blocked by an obstacle on the cellulose surface. Hence the cellobiohydrolases accumulates as an unproductive complex and the rate of the reaction decreases along the reaction time. That is particularly problematical for the hydrolysis of lignocellulosic materials which have several components attached to the cellulose. Thus, as the TrCel7A dissociation constant from cellulose is related to the extension and flexibility of the exo-loop covering the active site, a more open catalytic site could be a criterion for design of cellobiohydrolases tailored for hydrolysis of lignocellulosic materials. Moreover, the combination of cellulases exhibiting high probability of endo-initiation and accessory enzymes working on components attached to the cellulose (like hemicellulases and laccases) may be an interesting strategy to favor the cellobiohydrolases action upon the lignocellulosic materials.
